# The Process of Translation and Cross‐Cultural Adaptation of Functional Assessment Tools for Dementia: A Systematized Review

**DOI:** 10.1002/hsr2.70289

**Published:** 2025-01-07

**Authors:** Freddie O'Donald, Clara Calia

**Affiliations:** ^1^ School of Health in Social Science University of Edinburgh Edinburgh UK; ^2^ Department of Clinical Psychology NHS Tayside Dundee UK

**Keywords:** cross‐cultural adaptation, dementia, functional activity, measures

## Abstract

**Background and Aims:**

A decline in function related to impairment in cognitive abilities is required for diagnosing dementia. Cultural diversity influences everyday functioning, suggesting that functional assessment tools need to be culturally dependent. Therefore, this systematized review aimed to explore the translation and cross‐cultural adaptation of functional assessment tools designed to support dementia diagnosis.

**Methods:**

A systematic search of five electronic databases (CINAHL Plus, EMBASE, PubMed/MEDLINE, PsycINFO) was conducted from inception until September 2023. Quality assessment criteria were then utilized to evaluate the process of cross‐cultural adaptation and psychometric properties of identified functional assessment tools.

**Results:**

Fifteen studies relating to adapted functional assessment tools in 11 languages were identified. It was found that less than half of these studies fully adhered to established guidelines for the translation and cross‐cultural adaptation of instruments. Regarding psychometric properties, while the internal consistency and reliability of included measures were generally strong, there was variability in evaluating other psychometric properties, notably structural validity, measurement error, and cross‐cultural validity.

**Conclusions:**

This review underscores the need for researchers and clinicians to follow standardized guidelines for translating and cross‐culturally adapting functional assessment tools for dementia and ensuring the comprehensive evaluation of psychometric properties in cross‐cultural settings. Researchers and clinicians should consider whether the psychometric properties and characteristics of an adapted functional activity measure are suitable for use in their population of interest.

## 1 Introduction

Considering projected trends in population ageing, it is forecast that the prevalence of dementia is expected to increase worldwide [1]. The prevalence distribution is expected to be notably higher in low and middle‐income countries (LMICs) than in higher‐income countries [2, 3, 4]. In response to this, achieving a timely and accurate diagnosis of dementia has become a priority at the global level [5, 6, 7]. However, a substantial challenge in achieving accurate and timely diagnosis is limitations associated with the linguistic and cultural validation of existing assessment tools [8, 9, 10].

A core feature in the diagnosis of dementia is the identification of cognitive or behavioral symptoms that interfere with the ability to function during everyday activities [11]. Therefore, assessing functional ability is considered integral for accurate diagnosis and provision of clinical care [12, 13]. To date, there remains a lack of a gold standard for the assessment of functional activity in dementia [14, 15, 16, 17]. One approach is designing self‐report or observer‐based measures that evaluate the functions known to be impacted by AD [12]. However, the majority of these tests have been developed and validated in high‐income countries using the English language [14, 15, 19, 28]. This may have implications for achieving accurate and timely diagnosis in communities beyond which these assessments were initially developed, such as LMICs and migrant populations [10, 18, 20, 21].

Functional activity encompasses a broad range of tasks, such as managing finances, meal preparation, and communication, which have been shown to vary cross‐culturally [22, 23, 24, 25]. As the majority of functional assessment tools have been developed in high‐income countries, efforts have been made to adapt these measures for application across diverse cultural contexts [18, 19, 26]. The primary aim of such adaptations is often to maintain content equivalence, ensuring comparability between the original and adapted measures [27]. This necessitates a standardized approach to the adaptation of assessment tools developed in one context for use in other cultural settings [28, 29, 30, 31, 32, 33]. However, the degree to which assessments of functional activity have been adapted in a standardized way for assessing functional decline in dementia remains uncertain.

Cross‐cultural adaptation refers to the systematic preparation of an assessment tool for use in a different cultural context, ensuring its equivalence with the original assessment tool [28, 30, 34, 35]. It is asserted that assessments lacking a thorough cross‐cultural adaptation process may prove unsuitable for use within different cultural contexts due to the risk of potentially introducing cultural bias [10, 36, 37]. For instance, a questionnaire designed in a Western cultural context might include questions that assume certain norms or values specific to that culture, making it less relevant or meaningful in a non‐Western cultural context. Guidance for the cross‐cultural adaptation of instruments has been established; however, these recommendations exhibit notable heterogeneity [38, 39, 40, 41]. This can lead to variations in the steps employed to guarantee cultural relevance and the absence of potential bias during the adaptation of a measure [8, 42]. Considering the increasing need for timely and accurate dementia diagnosis on a global scale, ensuring equivalence in functional assessment across different cultures can be seen as a priority [30, 43]. This ensures patients have an equal chance of receiving the right diagnosis through the use of accurate and culturally relevant assessment measures.

This systematized review aims to investigate the current methodologies employed for the translation and cross‐cultural adaptation of functional assessment tools at a global level. Given their role in objectively assessing functional abilities, focusing on these tools is vital for informing accurate diagnosis of dementia across diverse cultural contexts. The guiding framework for this review is the Consensus‐based Standards for the Selection of Health Measurement Instruments (COSMIN) initiative, which has established criteria for evaluating psychometric properties across various domains [44, 45]. Our principal aim was to conduct a descriptive synthesis elucidating the quality of translation and cross‐cultural adaptation processes. Furthermore, we undertake a critical appraisal of the psychometric properties associated with these adapted measures. This work builds on previous studies, such as Yemm et al. [19], by conducting a more focused review of available cross‐culturally adapted functional assessment tools and their measurement properties.

## 2 Methods

This review has been reported in accordance with the Preferred Reporting Items for Systematic Reviews and Meta‐Analysis (PRISMA) guidelines [46]. The protocol for this review has been registered and prospectively published on the PROSPERO International Prospective Register of Systematic Reviews [CRD42023387459].

### 2.1 Eligibility Criteria

Included in this review were studies with a primary objective of translating and cross‐culturally adapting a functional assessment tool, coupled with an assessment of the psychometric properties of the adapted measure. Exclusive consideration was given to studies focusing on measures designed to support a diagnosis of dementia. This review encompasses both self‐reported, informant‐based, and objectively measured tools.

#### 2.1.1 Inclusion Criteria

The following inclusion criteria were applied, and studies were required to meet all criteria for inclusion:

- Studies reporting on the translation and cross‐cultural adaptation of functional assessment tools within a cultural context outside which the original measure was developed.
- Studies that translated and cross‐culturally adapted functional assessment tools known to support a dementia diagnosis. This was determined by the authors by assessing whether the original measure was explicitly intended for use in dementia care and whether it underwent validation within a population with cognitive decline.
- Studies that considered the psychometric properties of the adapted measure within a cultural context outside of which the original measure was developed.
- Studies that evaluated functional activity in people aged 50 years or older were either diagnosed with dementia or undergoing assessment for cognitive decline.
- Studies in all available languages and years were identified through searches in the undermentioned databases.

In accordance with the aims of this review, these criteria were employed to assess papers that report on the translation and cross‐cultural adaptation of functional assessment tools, alongside considering the psychometric properties of these adapted measures. The age criterion of 50 years was chosen as it corresponds to the timeframe in which prodromal dementia symptoms may begin to manifest, and assessment of functional activity is required [47, 48, 49, 50]. Furthermore, studies that measured both cognition and functional outcomes in a single tool were also considered for inclusion.

#### 2.1.2 Exclusion Criteria

The exclusion criteria for this review comprised: (i) Studies focusing on functional assessment tools intended for populations unrelated to dementia; (ii) Studies validating a functional assessment tool within its original cultural context; (iii) Studies that did not incorporate any statistical assessments of the adapted measure.

### 2.2 Information Sources

The following medical databases were searched: CINAHL Plus, EMBASE, PubMed/MEDLINE, and PsycINFO. No restrictions were placed on the search by language, geographical region, or lower date limit. The search was conducted on the 12 September 2023. In adherence to transparency, we acknowledge a deviation from the initial PROSPERO protocol, where backwards reference searching was planned but not conducted due to time constraints and prioritization of key databases.

### 2.3 Search Strategy

The Population Intervention Comparator Outcome (PICO) criteria were used to devise related search terms [51]. A combination of Medical Subject Headings (MeSH) and free text search terms related to functional activity, dementia assessment, and cross‐cultural adaptation were used in combination for the search strategy. The following search terms were utilized across the databases: (cultural adaption, cross‐cultural adaptation) AND (activities of daily living, ADL*, functional activity, instrumental activities of daily living, IADL*, functional abilit*) AND (assess*, questionnaire, scale, evaluat*, measure) AND (dementia). Further details on the search strategy are included in the Supplementary Material.

### 2.4 Study Selection

All studies identified from the electronic database search were imported into Covidence for automatic deduplication. Two independent reviewers then manually conducted screening and study selection within the Covidence platform. The reviewers independently screened records by title and abstract. Studies provisionally considered to meet the criteria for this review had their full text obtained and were read in full by the two reviewers to consider eligibility for inclusion. Any disagreements were resolved through discussion with a third reviewer, and Cohen's kappa was employed to establish the level of agreement between reviewers. Reasons for excluding a study after full‐text review were recorded and are detailed in the supplementary material.

### 2.5 Data Extraction Procedures

Data were extracted on the characteristics of included functional assessment tools and their measurement properties per COSMIN guidance [44]. Additionally, information regarding the translation and cross‐cultural adaptation procedures employed in each study was extracted. Data were extracted by a single reviewer, and a subset of papers (20%) was randomly selected for independent data extraction by a second reviewer.

For each study, the data extracted included the year of publication, country of origin, study authors, functional assessment tool name, the purpose of adaptation, the expertise of the committee adapting the measure, the cross‐cultural adaptation process, and psychometric properties. Regarding missing data or uncertainties, study authors were contacted for further information up to a maximum of two attempts. If no response was received, data extraction was completed using the information available.

### 2.6 Assessment of Methodological Quality

The methodological quality of each included study was evaluated according to two sets of guidance selected to align with the objectives of this review. Two independent reviewers evaluated the methodological quality of the included studies, resolving any disagreement through discussion.

*The Guidelines for the Translation and Cross‐Cultural Adaptations of Instruments* [28]. This guidance outlines a comprehensive process for the cross‐cultural adaptation of a measure, stipulating that this must include an initial translation, a synthesis of this translation, a back‐translation, reviews by an expert committee, and a pre‐test of the instrument. To evaluate the quality of the cross‐cultural adaptation in accordance with these guidelines, the criteria outlined by Costa et al. [52] were employed. These criteria, previously employed in other COSMIN reviews such as Albach et al. [53] and Praveen et al. [40], assign one of four ratings: (+) for a positive rating, (−) for a negative rating, (0) for no available information, and (?) for unclear information provided, for each stage of the adaptation process. This systematic approach ensures a robust evaluation aligned with the recommendations of Beaton et al. [28] and World Health Organization [41]. The steps and rating system are further outlined within the supplementary material.

*The Quality Assessment of Diagnostic Accuracy Studies Version 2* (QUADAS‐2), developed by Whiting et al. [54], was used as a tool for evaluating the potential risk of bias and applicability of studies included in this review. This tool encompasses four key domains: the method of participant selection, the use and interpretation of an index test, the use and interpretation of a reference standard, and the flow and timing of tests. The QUADAS‐2 thus offers a structured approach for evaluating the robustness and relevance of adapted measures in accurately assessing functional activity in dementia across these four domains.

### 2.7 Assessment of Psychometric Properties

The psychometric properties of included functional assessment tools were extracted and assessed against the COSMIN updated criteria for good measurement properties [44, 45]. These criteria are specified in Table 1.

**Table 1:** Criterion utilized to evaluate the psychometric properties of included measures of functional activity.

Measurement Property	Rating	Criteria
Structural Validity	**+**	*For Confirmatory Factor Analysis:* CFI, TLI, or alternative comparable indices > 0.95 **OR** RMSEA < 0.06 **OR** SRMR < 0.08
		*For Item Response Theory:* No violation of unidimensionality (CFI, TLI, or alternative comparable index > 0.95 **OR** RMSEA < 0.06 **OR** SRMR < 0.08)
		**AND**
		No violation of local independence (residual correlations among items after controlling dominant factor < 0.20)
		**AND**
		No violation of monotonicity (item scalability > 0.30)
		**AND**
		Adequate model fit
		IRT: *x* ^2^ > 0.01
		For Rasch: Infit and Outfit mean squares ≥ 0.5 and ≤ 1.5 **OR** Z standardized values > −2 and < 2.
	**?**	*For Confirmatory Factor Analysis:* Not all information for the “+” reported or this analysis is not conducted.
		*For Item Response Theory or Rasch Analysis:* Model fit not reported.
	**−**	Criteria for “+” not met.
Internal consistency	**+**	At least low evidence for sufficient structural validity **AND** Cronbach's alpha(s) ≥ 0.70 for the measure or subscales.
	**?**	Criteria for “At least low evidence for sufficient structural validity” not met **OR** This form of analysis is not conducted
	**−**	At least low evidence for sufficient structural validity **OR** Cronbach's alpha(s) < 0.70 for the measure or subscales.
Reliability	**+**	ICC or weighted Kappa ≥ 0.70
	**?**	ICC or weighted Kappa not reported **OR** this form of analysis is not conducted
	**−**	ICC or weighted Kappa < 0.70
Measurement Error	**+**	SDC or LoA < MIC
	**?**	MIC not defined
	**−**	SDC or LoA > MIC
Hypothesis Testing for Construct Validity	**+**	Result is in accordance with the hypothesis
	**?**	No hypothesis defined (by the review team)
	**−**	Result is not in accordance with the hypothesis
Cross‐Cultural Validity	**+**	No differences found between group factors (such as age, gender, or language) in multiple group factor analysis **OR** no important DIF for group factors (McFadden's *R* ^2^ < 0.02)
	**?**	No multiple group factor analysis **OR** DIF analysis performed
	**−**	Significant differences are observed between group factors **OR** DIF was found.
Criterion Validity	**+**	Correlation with gold standard measure ≥ 0.70 **OR** AUC ≥ 0.70.
	**?**	Not all information for “+” reported **OR** This form of analysis is not conducted.
	**−**	Correlation with gold standard measure < 0.70 **OR** AUC < 0.70
Responsiveness	**+**	The result is in accordance with the hypothesis **OR** AUC ≥ 0.70
	**?**	No hypothesis defined (by the review team)
	**−**	The result is not in accordance with the hypothesis **OR** AUC < 0.70

Abbreviations: AUC, area under the curve; CFI, comparative fit index; DIF, differential item functioning; ICC, intraclass correlation; LoA, limits of agreement; MIC, minimal important change; RMSEA, root mean square error of approximation; SDC, smallest detectable change; SRMR, standardized root mean square residual; TLI, Tucker–Lewis Index.

#### 2.7.1 The COSMIN Updated Criteria for Good Measurement Properties

Within these criteria, content validity is omitted, aligning with the conventional understanding that content validity holds particular significance during the development of original instruments [28]. Given that cross‐cultural adaptation often involves a review by an expert committee, content validity is implicitly assessed during this phase [55]. Subsequently, it becomes necessary to measure additional psychometric properties post‐adaptation [56].

### 2.8 Data Synthesis Methods

Following the extraction of data, the reviewers conducted a two‐step descriptive synthesis of the included studies. The first step included the assessment of the quality of the cross‐cultural adaptation process for the identified adapted functional assessment tools. Second, the psychometric properties of each adapted measure were examined and assessed against COSMIN criteria [44, 45]. The reviewers used no specific criteria to restrict the synthesis to a subset of studies and adopted no minimum criteria under which the data were synthesized.

## 3 Results

### 3.1 Results of the Search

Initial searches retrieved 567 articles from the electronic databases. After the removal of six duplicates, 561 articles were identified for title and abstract screening. Of these, 519 articles were excluded as they did not report on the translation and cross‐cultural adaptation of functional assessment tools designed to support a dementia diagnosis. Two authors then assessed 42 full‐text studies, which led to the further exclusion of 27 studies based on the eligibility criteria of this review. This resulted in the inclusion of 15 studies. The Cohen's kappa value of agreement between the reviewers was 0.96, with any disagreement resolved through discussion.

#### 3.1.1 PRISMA Flow Diagram

**Figure 1:**
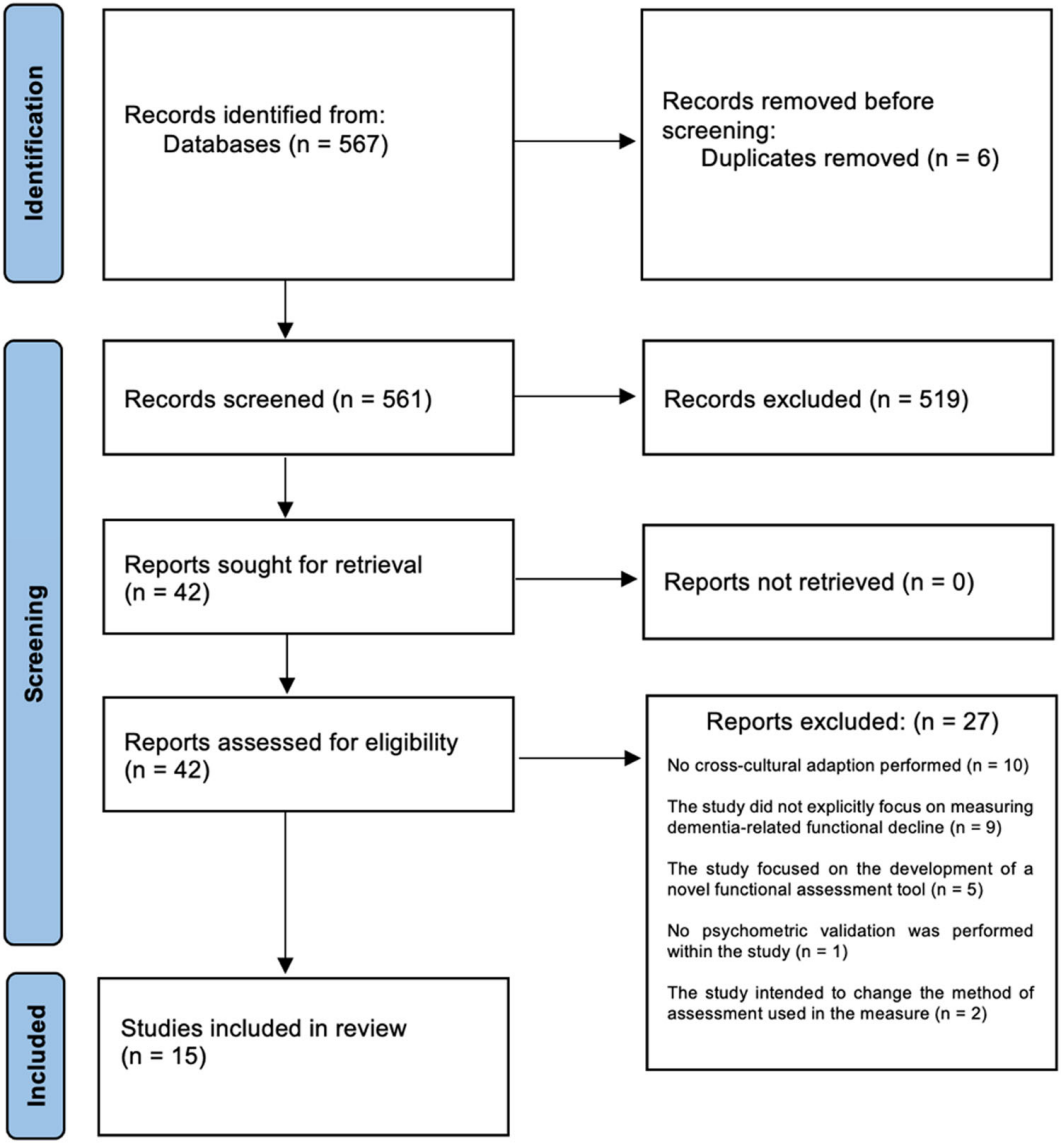
PRISMA flow diagram.

The PRISMA flow diagram outlines the identification, screening, and study selection process in this review. This is shown in Figure 1. Further details on the studies excluded during the full‐text review are available in the Supporting Information.

### 3.2 Characteristics of Included Studies

**Figure 2:**
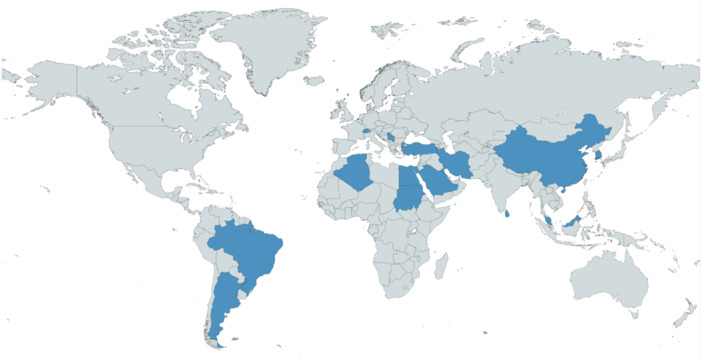
Heat map of locations included within this review, indicated by blue coloring.

This review included a total of 15 studies examining cross‐culturally adapted functional assessment tools, evaluating instruments in 11 different languages. All articles were published between 2003 and 2022, and the countries represented in this review are detailed in Figure 2. All studies were published in the English language.

An overview of the characteristics of the adapted functional assessment tools included in this review is detailed in Table 2. Most measures were cross‐culturally adapted by a panel of specialists with expertise in dementia or occupational therapy in consultation with linguistics experts.

**Table 2:** The Characteristics of Adapted Functional Assessment Tools.

Author(s) and year	Name of functional assessment tool	Method of assessment	Purpose of adaptation	Expertise of the committee adapting the measure	Language
Alkeridy et al. [57]	Bristol Activity of Daily Living Scale (BADLS)	Informant‐based	To translate and cross‐culturally adapt the BADLS for the Arabic‐speaking populations across the Middle East and Northern Africa, for application with dementia patients.	Researchers and clinicians with qualifications in medicine and geriatrics.	Arabic
Bruderer‐Hofstetter et al. [58]	The Amsterdam IADL Questionnaire Short Version (A‐IADL‐Q‐SV)	Informant‐based	To cross‐culturally adapt the A‐IADL‐Q‐SV for the German‐speaking population of Switzerland, for application with people diagnosed with MCI and dementia.	Researchers, in collaboration with memory clinic clinicians, specialists in medical translation, and informants of individuals diagnosed with dementia.	German
Choi et al. [59]	The Bayer‐activities of daily living (B‐ADL) instrument	Informant‐based	To translate and cross‐culturally adapt the B‐ADL for Korean speakers for use in detecting mild to moderate dementia.	Healthcare professionals in neurology, along with and two dementia research groups.	Korean
Chu and Chung [60]	The Activities of Daily Living Questionnaire (ADLQ)	Informant‐based	To cross‐culturally adapt the ADLQ measure for use with older adults of Hong Kong Chinese descent diagnosed with dementia.	A range of health professionals collaborating with family caregivers.	Chinese
Cintra et al. [61]	Alzheimer's Disease Cooperative Study – Activities of Daily Living Scale (ADCS‐ADL)	Informant‐based	To translate and cross‐culturally adapt the ADCS‐ADL for use as a measure of functional activity in Brazil, supporting the diagnosis of MCI and Alzheimer's Disease.	Dementia researchers adhering to a protocol for guiding the translation and cross‐cultural adaption of assessment tools.	Brazilian Portuguese
Gleichgerrcht et al. [62]	The Activities of Daily Living Questionnaire (ADLQ)	Informant‐based	To cross‐culturally adapt the ADLQ for Spanish speakers to assess functional activity across various types of dementia.	The development team comprised a dementia expert and a Spanish translator. Although the original ADLQ author was involved, the extent of their contribution remained unclear.	Spanish
Mehraban et al. [63]	The Lawton IADL Scale	Self‐report	To cross‐culturally adapt the Lawton IADL Scale for patients with dementia in Iran.	Occupational therapists and medical translators fluent in English and Persian.	Persian
Isik et al. [64]	The Lawton IADL Scale	Self‐report	To cross‐culturally adapt the Lawton IADL Scale for Turkish speakers for potential application with older adults experiencing cognitive decline.	Researchers fluent in both Turkish and English, collaborating with an expert committee.	Turkish
Medeiros and Guerra [65]	The Activities of Daily Living Questionnaire (ADLQ)	Informant‐based	To translate and cross‐culturally adapt the ADLQ into Brazilian Portuguese for measuring functional activity in patients with Alzheimer's disease.	A researcher, two health professionals, and bilingual educators collaborated on the development of the measure.	Portuguese
Kadar et al. [66]	The Lawton IADL Scale	Self‐report	To translate and cross‐culturally adapt the Lawton IADL Scale for application among elderly Malay speakers in Malaysia, with the aim of potential use in dementia patients.	Dementia researchers and occupational therapists specializing in elderly care.	Malay
Mello et al. [67]	The Routine Task Inventory – Extended (RTI‐E)	Direct Observation	To cross‐culturally adapt the RTI‐E for use in Brazilian Portuguese among older people with dementia.	Occupational therapists and translators fluent in English and Brazilian Portuguese.	Brazilian Portuguese
Milošević et al. [68]	The Amsterdam IADL Questionnaire Short Version (A‐IADL‐Q‐SV)	Informant‐based	To cross‐culturally adapt the A‐IADL‐Q‐SV for Serbian‐speaking patients attending memory clinics.	Dementia researchers and translators, collaborating with the original scale developer.	Serbian
Pereira et al. [69]	The Direct Assessment of Functional Status‐Revised (DAFS‐R)	Direct Observation	To translate and cross‐culturally adapt the DAFS‐R into Brazilian Portuguese for use in MCI and dementia.	A multidisciplinary panel of geriatric psychiatrists and neuropsychologists.	Brazilian Portuguese
Sanchez, Correa, and Lourenco [71]	The Functional Activities Questionnaire (FAQ)	Informant‐based	To cross‐culturally adapt the FAQ for use in Brazil for potential application with older adults experiencing cognitive decline.	Professionals specializing in geriatrics and gerontology.	Portuguese
Siriwardhana et al. [70]	The Lawton IADL Scale	Self‐report	To cross‐culturally adapt the Lawton IADL Scale from English to Sinhala for potential use among older adults with suspected dementia.	Researchers with expertise in public health and community medicine.	Sinhala

### 3.3 Quality of Cross‐Cultural Adaptation

The assessment of the quality of cross‐cultural adaptation for each study included is provided in Table 3. Overall, these findings consistently reflect positive evaluations for the translation and expert committee review stages, with 14 studies garnering positive ratings. Choi et al. [59] received a negative rating for translation quality because they employed only one translator, which may have compromised the accuracy and reliability of the translated materials, and Gleichgerrcht et al. [62] received an unclear rating due to the limited information available on the expert committee. Additionally, all included studies underwent pre‐testing within their intended population of use.

**Table 3:** The assessment of the quality of translation and cross‐cultural adaptation for functional assessment tools included in this review.

Functional Activity Measure	Language	Translation	Synthesis	Back Translation	Expert Committee Review	Pretesting
BADLS [57]	English to Arabic	+	+	−	+	+
A‐IADL‐Q‐SV [58]	Dutch to German	+	+	+	+	+
B‐ADL [59]	English to Korean	−	0	−	+	+
ADLQ [60]	English to Chinese	+	0	+	+	+
ADCS‐ADL [61]	English (US) to Brazilian Portuguese	+	+	+	+	+
ADLQ [62]	English to Spanish	+	?	+	?	+
Lawton IADL Scale [63]	English to Persian	+	+	−	+	+
Lawton IADL Scale [64]	English to Turkish	+	+	+	+	+
ADLQ [65]	English to Portuguese	+	0	−	+	+
Lawton IADL Scale [66]	English to Malay	+	+	+	+	+
RTI‐E [67]	English (US) to Brazilian Portuguese	+	+	+	+	+
A‐IADL‐Q [68]	English to Serbian	+	+	+	+	+
DAFS‐R [69]	English (US) to Brazilian Portuguese	+	+	−	+	+
FAQ [71]	English to Portuguese	+	0	+	+	+
Lawton IADL Scale [70]	English to Sinhala	+	+	+	+	+

**+** positive rating; *−* negative rating; 0 no information available; ? unclear.

Regarding back translation, ten studies met the criteria outlined in the guidelines for the Translation and Cross‐Cultural Adaptations of Instruments, while five received negative ratings. Among these, Alkeridy et al. [57], Choi et al. [59], Medeiros and Guerra [65], and Pereira et al. [69] each utilized a single independent translator for back translation. Mehraban et al. [63] received a negative rating because they did not employ a back translation procedure. In ten studies, a synthesis stage was employed to produce a consensus of the translations. In contrast, Choi et al. [59], Chu and Chung [60], Gleichgerrcht et al. [62], Medeiros and Guerra [65], and Sanchez et al. [71] exhibited variability or provided no information in their use of a synthesis stage, resulting in indeterminate ratings.

The best‐performing studies which met all criteria were Bruderer‐Hofstetter et al. [58], Cintra et al. [61], Isik et al. [64], Kadar et al. [66], Mello et al. [67], Milošević et al. [68], and Siriwardhana et al. [70]. Those at greatest risk of cultural bias for not reporting or following the guidelines for the Translation and Cross‐Cultural Adaptations of Instruments were Choi et al. [59], Gleichgerrcht et al. [62], and Medeiros and Guerra [65].

### 3.4 Psychometric Properties of Adapted Functional Assessment Tools

The ratings of measurement properties per language and tool, as assessed using the COSMIN updated criteria for good psychometric properties [44, 45], are presented in Table 4. Further details of the psychometric properties of included measures are presented in Table 5.

**Table 4:** Ratings of Measurement Properties for Adapted Measures Using COSMIN Criteria for Good Measurement Properties.

Functional assessment tool	Structural validity	Internal consistency	Reliability	Measurement error	Hypothesis testing for construct validity	Cross‐cultural validity	Criterion validity	Responsiveness
BADLS [57]	?	+	?	?	+	?	+	+
A‐IADL‐Q‐SV [58]	+	?	+	+	+	+	−	+
B‐ADL [59]	?	+	+	?	+	−	+	+
ADLQ [60]	?	+	+	?	+	+	+	+
ADCS‐ADL [61]	?	+	?	?	+	?	+	+
ADLQ [62]	?	+	+	?	+	?	−	+
Lawton IADL Scale [63]	?	?	+	?	+	?	−	+
Lawton IADL Scale [64]	?	+	+	?	+	?	+	+
ADLQ [65]	?	+	?	?	+	?	+	+
Lawton IADL Scale [66]	?	+	+	?	?	?	?	?
RTI‐E [67]	?	+	+	?	+	?	?	+
A‐IADL‐Q [68]	?	+	+	?	+	?	+	+
DAFS‐R [69]	?	+	+	?	+	?	−	+
FAQ [71]	?	+	+	?	?	?	?	?
Lawton IADL Scale [70]	+	+	+	?	+	?	−	+

+ positive rating; − negative rating; ? indeterminate rating.

**Table 5:** The psychometric properties of included measures.

Functional assessment tool	Structural validity	Internal consistency	Reliability	Measurement error	Hypothesis testing for construct validity	Cross cultural validity	Criterion validity	Responsiveness
BADLS [57]	**NR**	α = 0.95	**NR**	**NR**	Result in accordance with hypothesis.	**NR**	Spearman correlation coefficients were *r* = *−0.86* for the *Arabic BADLS* and *Arabic Katz ADL*, and *r* = *−0.82* for *Arabic BADLS* and the *MOCA*.	Result in accordance with the hypothesis.
		Item‐Total correlations found to be significant.						
A‐IADL‐Q‐SV [58]	CFI = 0.95	**NR**	ICC = 0.93	SDC of 6.6 < MIC of 7.9	Result in accordance with hypothesis.	Differences in McFadden's pseudo‐R‐squared values for group factors were minimal, with R‐squared values less than 0.02.	Spearman correlation coefficients of the *German A‐IADL‐Q‐SV* with the *IQCODE* were r = −0.69 and were r = −0.41 for the *German A‐IADL‐Q‐SV* with the *Lawson‐Brody IADL* scale.	Result in accordance with the hypothesis.
	RMSEA = 0.11							A Kruskal‐Wallis rank sum test showed significant differences between the MCI group and cognitively healthy participants.
B‐ADL [59]	PCA yielded a single‐factor solution explaining 70% of the variance (eigenvalue = 17.5), with factor loadings ranging from 0.73 to 0.93.	α = 0.98	ICC = 0.95	**NR**	Result in accordance with hypothesis	**NR**	Pearson correlation coefficients were *r* = −0.77 for *Korean B‐ADL* and *MMSE*, and *r_=* 0.7 for *Korean B‐ADL* and *SKT*.	Result in accordance with the hypothesis.
	CFA not conducted.	All Individual component loadings > 0.7	Spearman correlation coefficients for items ranged between 0.52 and 0.95 between the test‐and retest.			Multiple linear regression showed gender, age, and education had no significant influence on B‐ADL scores.		AUC = 0.906
ADLQ [60]	PCA analysis identified a six‐component solution, accounting for 85.4% of total variance. All Individual component loadings were > 0.7	α = 0.81	ICC = 0.997	**NR**	Result in accordance with hypothesis.	DIF not conducted.	Pearson correlation coefficients were r = −0.91 for the *Chinese ADLQ* with the *CDAD*. For the *Chinese ADLQ* with the *MMSE* the correlation coefficient was r = −0.79	Result in accordance with the hypothesis.
	CFA not conducted.	All Individual component loadings > 0.7				No significant differences found between group factors: age (*p* = 0.12), gender (*p* = 0.56), and language (*p* = 0.22).		
ADCS‐ADL [61]	**NR**	α = 0.89	**NR**	**NR**	Result in accordance with hypothesis.	**NR**	The correlation between the *Brazilian Portuguese ADCS‐ADL* and the *Pfeffer IAQ* was found to be 0.89	Result in accordance with the hypothesis.
		Use of cross‐validation to examine if subsets of the scale demonstrate accuracy in assessing cognitive status.						AUC = 0.89
ADLQ [62]	EFA with varimax rotation identified a six‐factor solution explaining 81.6% of variance. Items were loaded > 0.4 for all items.	α = 0.88	Cohen's k was 0.90	**NR**	Result in accordance with hypothesis.	**NR**	Spearman correlation coefficients of the *Spanish ADLQ* with the *FAQ* = 0.67. For the *Spanish ADLQ* with the *CDR, r* = 0.54.	Result in accordance with the hypothesis.
		Factor structure matches those proposed in the original ADLQ.						
	CFA not conducted.							
Lawton IADL Scale [63]	**NR**	The Cronbach's alpha values after item deletion ranges between 0.728 and 0.768 for each item on the scale.	ICC = 0.993	**NR**	Result in accordance with hypothesis.	**NR**	The Pearson correlation coefficient was r = −0.688 for the *Persian Lawton IADL Scale* with the *FAST*.	Results in accordance with the hypothesis and comparable to other adapted Lawton IADL scales.
	High level of agreement between experts on content validity.	No total Cronbach alpha coefficient presented.						
Lawton IADL Scale [64]	**NR**	α = 0.843	ICC = 0.915	**NR**	Result in accordance with hypothesis.	**NR**	Pearson correlation coefficients were r = 0.896 for the *Turkish Lawton IADL Scale* with the *Katz Index*. For the *Turkish Lawton IADL Scale* with the *FIS* the correlation coefficient was r = 0.850	Result in accordance with hypothesis.
		Internal consistency was assessed for item of the scale. Values ranged from 0.806 to 0.850						
ADLQ [65]	EFA with varimax rotation identified a six‐factor solution.	α = 0.759	**NR**	**NR**	Result in accordance with hypothesis.	**NR**	The Pearson correlation coefficient between the *Portuguese ADLQ* and the *MMSE was −0.79*	Result in accordance with hypothesis.
	CFA not conducted.	Factor structure matches that proposed in the original measure.						
Lawton IADL Scale [66]	**NR**	α = 0.838	ICC = 0.957	**NR**	No hypothesis defined.	**NR**	**NR**	No hypothesis defined.
		Content validity assessment indicated high CVI scores.					No gold standard comparator measure used in study.	
RTI‐E [67]	**NR**	α = 0.813	ICC = 0.987	**NR**	Result in accordance with hypothesis.	**NR**	**NR**	Result in accordance with hypothesis.
		Self‐report and informant reports were compared to assess the structural validity of the adapted measure. Both methods exhibited similar α values across each scale of the RTI‐E.					No gold standard comparator measure used in study.	
A‐IADL‐Q [68]	**NR**	α = 0.82	ICC = 0.92	**NR**	Result in accordance with hypothesis.	**NR**	The Kendall tau‐b correlation coefficient between the *Serbian A‐IADL‐Q* and the *LB‐IADL* was determined to be 0.714, while for the *Serbian A‐IADL‐Q* and the *MMSE*, a correlation coefficient of 0.638 was observed.	Result in accordance with hypothesis.
		The *Serbian A‐IADL‐Q* demonstrates comparable explanatory power to the original version when correlated with MMSE scores, indicating its structural validity in assessing activities of daily living.						
DAFS‐R [69]	**NR**	α = 0.78	ICC = 0.996	**NR**	Result in accordance with hypothesis.	**NR**	Pearson correlation coefficient between the *Portuguese DAFS‐R* and *IQCODE* = −0.65	Result in accordance with hypothesis.
		Item‐Total correlations found to be significant.						AUC = 0.998
FAQ [71]	**NR**	α = 0.95	ICC = 0.97	**NR**	No hypothesis defined.	**NR**	**NR**	No hypothesis defined.
		Evidence of high content validity.						
Lawton IADL Scale [70]	CFI = 0.98	α = 0.91	ICC = 0.74	**NR**	Result in accordance with hypothesis.	**NR**	Spearman correlation coefficients of the *Sinhala Lawton IADL* with the *Barthel Index* = 0.61. For the *Sinhala Lawton IADL* with the *MOCA, r* = 0.41	Result in accordance with hypothesis.
	SRMR = 0.06							

Abbreviations: ADCS‐ADL, Alzheimer's disease cooperative study activities of daily living inventory; AUC, area under the curve; CDAD, Chinese version of the disability assessment for dementia; CFA, confirmatory factor analysis; CFI, Comparative Fit Index; DAFS‐R, direct assessment of functional status‐revised; DIF, differential item functioning; EFA, exploratory factor analysis; FAQ, functional activities questionnaire; FAST, Functional Assessment Staging Tool; FIS, Functional Independence Score; ICC, Intraclass Correlation Coefficient; IQCODE, informant questionnaire on cognitive decline in the elderly; LB‐IADL, Lawton–Brody Instrumental Activities of Daily Living; MCI, mild cognitive impairment; MIC, minimal important change; MMSE, Mini‐Mental State Examination; MOCA, Montreal Cognitive Assessment; PCA, principal component analysis; NR, not reported; Pfeffer IAQ, Pfeffer Functional Activities Questionnaire; RMSEA, Root Mean Square Error of Approximation; SDC, smallest detectable change; SKT, short cognitive performance test; SRMR, standardized root mean square residual.

Our review of psychometric properties among the examined measures revealed considerable variability in measurement properties. Structural validity garnered positive ratings for 2 measures, while evaluations of this construct were absent for 13 measures. Reliability demonstrated favorable results across various functional activity measures, with 12 earning positive ratings. Internal consistency and Hypothesis testing for construct validity received positive ratings in 14 measures. However, evaluations for measurement error and cross‐cultural validity were limited, with only 1 measure receiving positive ratings for measurement error and 2 for cross‐cultural validity. Criterion validity showed positive ratings in 7 measures, while positive responsiveness was observed in 13.

These findings reveal generally positive trends in psychometric properties for adapted functional assessment tools, with notable strengths in internal consistency, reliability, and hypothesis testing for construct validity.

### 3.5 Methodological Quality Assessment of Included Studies

The assessment of the methodological quality of included studies with the QUADAS‐2 is presented in Table 6. All studies, regardless of their assessed quality, were included to demonstrate the full availability of culturally adapted functional assessment tools identified by this review.

**Table 6:** The methodological quality of included studies.

	Risk of bias				Applicability		
	Patient selection	Index test	Reference standard	Flow and timing	Patient selection	Index test	Reference standard
Alkeridy et al. [57]	**−**	**+**	**−**	**−**	**−**	**−**	**−**
Bruderer‐Hofstetter et al. [58]	**−**	**−**	**−**	**−**	**−**	**−**	**−**
Choi et al. [59]	**−**	**−**	**−**	**−**	**−**	**−**	**−**
Chu and Chung [60]	**−**	**?**	**−**	**−**	**−**	**−**	**−**
Cintra et al. [61]	**−**	**−**	**−**	**?**	**−**	**−**	**−**
Gleichgerrcht et al. [62]	**+**	**−**	**−**	**+**	**+**	**−**	**−**
Mehraban et al. [63]	**−**	**−**	**−**	**−**	**−**	**+**	**−**
Isik et al. [64]	**?**	**?**	**−**	**?**	**−**	**−**	**−**
Medeiros and Guerra [65]	**−**	**+**	**+**	**−**	**−**	**?**	**−**
Kadar et al. [66]	**+**	**−**	**?**	**−**	**+**	**−**	**?**
Mello et al. [67]	**−**	**−**	**−**	**−**	**−**	**−**	**−**
Milošević et al. [68]	**−**	**−**	**−**	**−**	**−**	**−**	**−**
Pereira et al. [69]	**−**	**−**	**−**	**−**	**+**	**−**	**−**
Sanchez, Correa, and Lourenco [71]	**−**	**−**	**?**	**−**	**−**	**−**	**−**
Siriwardhana et al. [70]	**+**	**−**	**−**	**?**	**−**	**−**	**−**

**+** = high risk, − = low risk, ? = unclear risk, green = low risk, red = high risk, yellow = unclear risk.

The inter‐rater reliability for QUADAS‐2 ratings, as evidenced by Cohen's weighted kappa (*k*) value of 0.869, reflects a high level of agreement between raters. A consensus was reached on 100% of occasions when disagreements arose.

Overall, the risk of bias in patient selection was considered acceptable, with 11 studies rated as low risk and 1 rated as unclear. However, 3 studies—Gleichgerrcht et al. [62], Kadar et al. [66], and Siriwardhana et al. [70]—were identified as having a high risk of bias within this domain. Regarding the risk of bias associated with the use of an appropriate index test, 11 studies were rated as low risk, while 2 were rated as unclear. Notably, Alkeridy et al. [57] and Medeiros and Guerra [65] received high‐risk ratings. In evaluating the risk of bias associated with the use of an appropriate reference test, 12 studies received low‐risk ratings, 2 were rated as unclear, and Medeiros and Guerra [65] were identified as high risk. Considering the risk of bias related to the flow and timing of assessment, 11 studies were rated as low risk, and 3 were rated as unclear. Additionally, one study received a high‐risk rating, namely, Gleichgerrcht et al. [62].

When assessing the applicability of measures using the QUADAS‐2 criteria, 12 studies exhibited a low level of concern in the patient selection domain, with this number increasing to 13 for the index test domain and 14 for the reference standard domain. In general, a higher proportion of studies raised concerns regarding applicability in the patient selection domain, with 3 receiving high‐risk ratings, compared to 1 in the index test domain and none in the reference standard domain. Medeiros and Guerra [65] received an unclear rating for applicability in the index test domain, whereas Kadar et al. [66] received an unclear rating in the reference standard domain.

The best‐performing studies for low risk of bias and applicability on the QUADAS‐2 which met all criteria were Bruderer‐Hofstetter et al. [58], Choi et al. [59], Mello et al. [67], and Milošević et al. [68]. Those at greatest risk were Gleichgerrcht et al. [62], Medeiros and Guerra [65], and Kadar et al. [66], which received poor ratings due to the potential for bias in the selection of patients, the reference standard and interpretation of the index test, all of which had the potential to introduce bias. Additionally, concerns arose that the patients included may not adequately represent the group the measure was originally designed for, which could affect its intended use.

## 4 Discussion

In this systematized review, we identified 15 studies that reported on the translation and cross‐cultural adaptation of functional assessment tools designed to facilitate a diagnosis of dementia. The primary objective was to evaluate the quality of translation and cross‐cultural adaptation of these measures, while a secondary aim was to critically appraise their measurement properties.

The findings of this review revealed that 46.7% (seven studies) adhered to established guidelines for the translation and cross‐cultural adaptation of instruments. Furthermore, it highlighted a notable gap, as none of the included studies reported a complete assessment of all measurement properties, as outlined by the updated COSMIN criteria for good psychometric properties. It is important to note that many of these studies were conducted before the introduction of the updated COSMIN criteria, which may account for their limited adherence. However, our findings underscore the need to promote the implementation of frameworks that facilitate both cross‐cultural adaptation and comprehensive evaluation of psychometric properties for functional assessment tools in dementia across various cultural backgrounds.

The World Health Organization recommends the translation and cross‐cultural adaptation of currently available instruments for use in different cultural contexts, thereby promoting parity among assessments at the international level [72]. The cross‐cultural adaptation process for functional assessment tools in dementia broadly adhered to the guidelines outlined in the Translation and Cross‐Cultural Adaptations of Instruments [28]. However, inconsistency was observed in the application of adaptation methodologies across studies. The implementation of a back‐translation procedure involving two independent translators emerged as the least frequently followed step in the cross‐cultural adaptation of measures included in this review. This finding is consistent with other COSMIN reviews investigating the cross‐cultural adaptation of different measurement constructs [40, 73]. Back‐translation is a relatively straightforward and effective method for enhancing the quality of cross‐cultural adaptation, preserving the original meaning of the measure, and providing insights into translation accuracy [74]. While its use depends on the availability of suitable translators, some researchers argue that an expert committee may provide more value than back‐translation alone [39], which may explain why this step is less frequently followed. However, when aiming to maintain content equivalence, combining back‐translation with the input of an expert committee ensures both linguistic accuracy and the retention of optimal wording in the target language, which is why these steps are emphasized in most guidelines for cross‐cultural adaptation.

In our review, we found that the majority of studies reported content modifications to the adapted measures. This commonly involved authors providing clarified examples of functional activities tailored to align with the cultural values of the population for which they were being adapted. For instance, Chu and Chung [60] adapted the recreation item of the ADLQ by replacing “bridge and golf” with “mahjong playing,” while Alkeridy et al. [57] revised the finances item by removing “writing a cheque” and substituting this with “recognize money value and can buy things.” The influence of the committee's expertise on content modification and the potential ramifications of variations in this process on cultural equivalence remains unclear, indicating a need for additional guidance to standardize the process [30, 39].

In the process of cross‐cultural adaptation of a measure, it is recommended to evaluate a range of psychometric properties, such as validity and reliability [75, 76, 77]. This review found that adapted functional assessment tools demonstrated strong internal consistency and reliability according to COSMIN criteria. The available information on structural validity and measurement error was limited. Specifically, only one study reported findings on measurement error and two studies on structural validity within the context of this review. Furthermore, a substantial portion of the studies did not evaluate cross‐cultural validity, resulting in only two studies meeting the COSMIN criteria for adequate cross‐cultural validity in this review. Additionally, the QUADAS‐2 highlighted the risk of bias in certain studies, such as Gleichgerrcht et al. [62] and Kadar et al. [66], due to concerns regarding sample representativeness and the appropriate application of index tests and reference standards. The inconsistency in validation methodology and potential risk of bias in some studies limits the evidence available to support the endorsement of specific adapted functional assessment tools for dementia diagnosis. Therefore, this review instead offers recommendations to address these gaps.

### 4.1 Recommendations for Future Research

In considering the future adaptation of functional assessment tools across different cultural contexts, this review highlights the advantages of incorporating standardized guidelines to ensure the quality of adaptation. Researchers engaging in the adaptation of functional assessment tools should explicitly outline the adaptation process within study methodologies. Given the heterogeneity in cross‐cultural adaptation guidelines [31, 38], this should promote the development of best practices.

The influence of the review committee's expertise on the process and the adaptation of content within measures remains uncertain. It would be advisable for review committees to evaluate the amount of overlap in content between measures, thus ensuring consistent understanding across languages and cultural groups. The involvement of dementia patients and caregivers in the adaptation process, using focus groups or interviews during this phase, might enhance the face validity of any adapted content [78]. This would be consistent with emerging toolkits, such as Strengthening Responses to Dementia in Developing Countries (STRiDE), which aims to develop and adapt assessment tools using rigorous methods to enhance dementia care at the global level [79]. However, the psychometric properties of such toolkits have yet to be reported.

While included studies demonstrated adequate reliability, there was variability in the assessment of other psychometric properties, such as measurement error and structural validity. It can be argued that converging evidence across multiple psychometric properties is required to establish the validity of an instrument within a different cultural context, as each contributes specific information about the quality of a measure [80, 81]. This highlights the importance of establishing a standardized approach for evaluating the psychometric properties of adapted functional assessment tools, with particular emphasis on assessing properties that have not yet been examined. Implementing a standardized approach to evaluation is likely to promote equity in the quality of functional assessment across different cultural contexts, reflecting a key priority in global dementia research [5]. Furthermore, considering measurement error within these studies, incorporating feedback from people with dementia and their caregivers regarding observed changes in functional activity across different cultural contexts would appear important for establishing thresholds for minimal important change [82, 83]. Additionally, employing longitudinal cohort designs that map functional decline across various cultural contexts would likely augment this perspective [84].

A key finding in this review was that the psychometric property of cross‐cultural validity was not assessed in the majority of included studies. Few studies examined variables such as education level or socioeconomic status within a culture, which can limit conclusions regarding the performance of the adapted functional assessment tool as a true reflection of the original scale [85]. Demonstrating cultural validity is complex and involves not only education level and socioeconomic status but also other factors such as age group, relationship status, religion, cultural norms, and stigma. These factors can significantly influence how a measure of functional ability is perceived and utilized across different cultural contexts. Many studies did not explicitly explore these dimensions, constraining our ability to assess the effectiveness of adapted tools across diverse populations. Future research should employ methodologies such as differential item functioning (DIF) to determine whether group‐specific factors affect responses and ensure that the adapted measures maintain equivalence with the original tools [86]. A broader consideration of these cultural factors will aid the development of tools that truly reflect the intended population and enhance their overall applicability.

### 4.2 Implications for Clinical Practice

The implications for clinical practice derived from this review are unclear. While the review emphasizes the reliability and responsiveness of adapted functional assessment tools as indicators of functional decline in dementia, there is a notable absence in the evaluation of other psychometric properties outlined by COSMIN. This absence in the evaluation of specific psychometric properties implies that employing a diverse range of tools may be essential for the accurate assessment of functional activity across different cultural contexts. Healthcare professionals should acknowledge the importance of adopting a context‐specific approach to assessment, as this can enhance the relevance of an assessment in capturing the subtleties of dementia‐related functional decline within different cultural groups.

In addition, institutions involved in the translation and cross‐cultural adaptation of instruments should provide clear guidance on the content modification process. This is especially important when adapting various functional activities to a new cultural context, requiring the involvement of an expert committee. Incorporating guidance from the World Health Organization [41] and the International Test Commission [31] may facilitate a standardized process for content modification. For instance, the use of translators and an expert committee who are native to the local culture and language during the adaptation process. However, efforts are needed to refine best practices further to balance the requirement for linguistic and cultural equivalence within adapted functional activity measures while also ensuring suitability for use within a different culture. This should involve clearly communicating any modifications made to an adapted instrument and the rationale behind such modifications while maintaining a commitment to a standardized approach.

### 4.3 Strengths and Limitations

This review is subject to certain limitations that must be considered. Firstly, the selection of studies may have been constrained by the search terms employed, potentially leading to the exclusion of relevant research. For instance, omitting the term “translation” from the search strategy was intended to identify studies that ensured both linguistic and cultural appropriateness across diverse populations. However, this approach may have excluded relevant studies that focused solely on translation. Additionally, we did not include search terms for specific dementia subtypes, such as “Alzhiemer*,” as our review aimed to cover all types of dementia. While the inclusion of MeSH terms for “dementia” should capture relevant studies, we recognize that this choice may have influenced our results. Furthermore, the absence of lateral searches, which could have helped identify additional articles, may also have led to the exclusion of relevant research. Any future reviews in this area could benefit from integrating backward reference searching to ensure the inclusion of all relevant studies.

Additionally, while the exclusion criteria of this review adhered to COSMIN guidance for evaluating measurement properties by excluding papers lacking analysis of psychometric properties, this may have limited the inclusion of studies providing insights into cross‐cultural adaptation processes. Furthermore, the review only searched in academic databases, and certain functional assessment tools are commercially distributed, undergoing their own cross‐cultural adaptation processes and quality assurance. As a result, these tools might not be documented in academic journals and could thus be excluded from this review.

Despite its limitations, this study highlights a significant concern: despite the widespread global use of functional assessment tools, only 15 studies were identified. This may underscore a larger issue surrounding the use of these tools without rigorous adaptation for diverse cultural contexts. Furthermore, this study provides valuable insights to assist future researchers in designing studies for the adaptation of functional assessment tools in dementia across various cultural settings. The quality assessment revealed that although some studies exhibited a risk of bias, the majority demonstrated moderate to good quality and applicability. Additionally, the review covered various functional activity measures across a diverse range of populations, indicating that a meta‐analysis could be useful for integrating findings from different studies to identify the most effective measures as more evidence accumulates.

## 5 Conclusions

In summary, this systematized review evaluated the psychometric properties and cross‐cultural adaptation processes of 15 adapted functional assessment tools for use in dementia. The findings underscore a significant gap in adherence to established guidelines, with only 46.67% of studies following recommended practices for the cross‐cultural adaptation of instruments and none conducting a comprehensive evaluation of psychometric properties per COSMIN criteria. Additionally, while most studies conducted psychometric evaluation concerning internal consistency, criterion validity, and reliability for an adapted measure, information related to measurement error, cross‐cultural validity, and structural validity was lacking. Future research involving the translation and cross‐cultural adaptation of functional activity measures should adhere to recommended guidelines to ensure equivalence in content between the adapted and original measures. Researchers and clinicians should ensure the use of multiple bilingual translators native to the local culture and language. Additionally, consideration should be given to the expertise of the panel in modifying the content of a measure during the cross‐cultural adaptation process. Furthermore, future work on adapting measures should incorporate all aspects of psychometric analysis recommended by COSMIN, ensuring both clinical effectiveness and cultural validity.

## Author Contributions

**Freddie O'Donald:** conceptualization, methodology, validation, formal analysis, investigation, writing, visualization, project administration. **Clara Calia:** methodology, validation, investigation, visualization, project administration.

## Disclosure

The authors confirm that no material from other sources has been reproduced in this work that requires permission for reproduction.

The lead author Freddie O'Donald affirms that this manuscript is an honest, accurate, and transparent account of the study being reported; that no important aspects of the study have been omitted; and that any discrepancies from the study as planned (and, if relevant, registered) have been explained.

## Conflicts of Interest

The authors declare no conflicts of interest.

## Supporting information

Supporting information.

## Data Availability

The data supporting this review are from publicly available databases and published studies. No new datasets were generated or analyzed.
