# Supplementary material for: The Process of Translation and Cross‐Cultural Adaptation of Functional Assessment Tools for Dementia: A Systematized Review
Source: Health Sci Rep. 2025 Jan 7;8(1):e70289. doi: 10.1002/hsr2.70289 (PMC11705501; doi:10.1002/hsr2.70289)
Supplement: Supplementary file 1 — Supporting information. [file HSR2-8-e70289-s001.docx]

**Supplementary Material for Systematic Review**

The supplementary material for this review presents the reasons for excluding studies at the full-text review stage, presented in *Supplementary Table 1.* Additionally, the assessment criteria for evaluating the quality of cross-cultural adaptation from Costa et al. (2009) is outlined in *Supplementary Table 2;* and the search strategies used are provided in *Supplementary Table 3.*

*Supplementary Table 1: Reasons for Excluded Studies*

| **Author(s) and Year** | **Reason for Exclusion** |
| --- | --- |
| (Larsen et al., 2021) | No psychometric validation was performed within the study. |
| (Collingwood et al., 2014)  (Yasunaga et al., 2007)  (Paula et al., 2014)  (Johansson et al., 2016)  (Quiroga, Albala, & Klaasen., 2004) | The study focused on the development of a novel functional assessment tool for older adults. |
| (Stringer et al., 2021)  (Sikkes et al., 2013) | The study intended to change the method of assessment used in the measure. |
| (Soler-König et al., 2016)  (Magnussen et al., 2010)  (Vaughan et al., 2013)  (Merellano-Navarro et al., 2015)  (Williams et al., 2012)  (Alegre-Muelas et al., 2019)  (Vergara et al., 2012)  (Law et al., 2014)  (Martínez-Martín et al., 2009) | The measure was not explicitly focused on measuring dementia-related functional decline in older adults. |
| (Ng et al., 2006)  (Umayal et al., 2010)  (Leung et al., 2011)  (Sánchez-Benavides et al., 2009)  (Harper et al., 2019)  (Tafiadis et al., 2023)  (Stacke et al., 2020)  (Kaur et al., 2016)  (Cabañero-Martínez et al., 2009)  (Tozlu et al., 2014) | No cross-cultural adaptation was performed in the study. |
|  | |

*Supplementary Table 2: Guidelines for the Process of Cross-Cultural Adaptation of Instruments*

| **Adaptation Step** | **Description** | **Rating** |
| --- | --- | --- |
| Translation | Two (or more) translators should independently translate the original instrument. The translators should preferably be native speakers of the target language. | **+** Translation performed by two or more independent translators. |
|  |  | **-.**Translation is performed by only one translator. |
|  |  | **?** Doubtful translation procedure. |
|  |  | **0** No information about translation. |
| Synthesis | The translators should synthesise the multiple translations to produce a consensus of the translations. | **+** Performed synthesis. |
|  |  | **?** Doubtful design. |
|  |  | **0** No information about synthesis OR translation performed by only one translator. |
| Back Translation | Translators, blinded to the original measure, should translate the consensus translation back into the original language. | **+** Back translation performed by at least two independent translators. |
|  |  | **-.**Only one translator performs back translation. |
|  |  | **?** Doubtful back translation procedure. |
|  |  | **0** No information about back translation. |
| Expert Committee Review | The expert committee should consolidate all the versions of the instrument and develop what would be considered the “prefinal” version of the measure for testing. | **+** Clearly reported the existence of an expert committee. |
|  |  | **?** Doubtful design. |
|  |  | **0** No information about the expert committee. |
| Pretesting | The “prefinal” instrument undergoes pilot testing with members of the target population. | **+** Performed pretesting. |
|  |  | **?** Doubtful design. |
|  |  | **0** No information on pretesting. |
| **+** = *positive rating*; **-** = *negative rating*; **0** = *no information available*; **?** = *unclear* | | |

*Supplementary Table 3: Example Search Strategy*

| *Search Strategy* | "Cultural Characteristics"[MeSH] OR "Cross-Cultural Comparison"[MeSH] OR "Transcultural Nursing"[MeSH] "Activities of Daily Living"[MeSH] OR "Functional Laterality"[MeSH] "Dementia"[MeSH] OR "Neurocognitive Disorders"[MeSH] (cultural adaptation OR cross-cultural adaptation) AND (activities of daily living OR ADL* OR functional activity OR instrumental activities of daily living OR IADL* OR functional abilit*) AND (assess* OR questionnaire OR scale OR evaluat* OR measure) AND (dementia) |
| --- | --- |
